# Estimation of shedding time in laboratory-confirmed COVID-19 cases in South Africa: a population-based record linkage study, March-December 2020

**DOI:** 10.11604/pamj.2023.46.24.41047

**Published:** 2023-09-15

**Authors:** Carroll Tshabane, Lazarus Kuonza, Hetani Mdose, Alfred Musekiwa, Nkengafac Villyen Motaze

**Affiliations:** 1 South African Field Epidemiology Training Programme, National Institute for Communicable Diseases, a Division of the National Health Laboratory Service, Johannesburg, South Africa,; 2 School of Public Health, Faculty of Health Sciences, University of Witwatersrand, Johannesburg, South Africa,; 3 School of Health Systems and Public Health, Faculty of Health Sciences, University of Pretoria, Pretoria, South Africa,; 4 Medicine Usage in South Africa, School of Pharmacy, Faculty of Health Sciences, North-West University, Potchefstroom, South Africa

**Keywords:** COVID-19, SARS-CoV-2, viral shedding time, South Africa, population-based

## Abstract

**Introduction:**

in South Africa, COVID-19 cases are notifiable and hospitalized cases are reported on a dedicated platform. It is crucial to estimate the duration of SARS-CoV-2 shedding to inform public health interventions. We aimed to estimate viral shedding time among laboratory-confirmed COVID-19 cases in South Africa.

**Methods:**

we analyzed COVID-19 PCR results from 5 March to 31 December 2020. We included cases with at least 2 consecutive positive PCR tests and a subsequent negative test. We performed multiple linear regression to determine the association between shedding time and predictor variables (age, sex, admission status and province). We included 2752 cases that met the inclusion criteria.

**Results:**

about 39.9% (1099/2752) of participants were inpatients and 60.1% (1653/2752) were outpatients. The median shedding time was 17 days (range: 1-128). There was no difference in shedding time between males and females and between hospitalized patients and outpatients. Individuals aged 0-4 years had the lowest shedding time (median: 14 days, range: 1-72). After adjusting for age, sex and province, shedding time was shorter for hospitalized patients compared to outpatients (co-efficient: -0.14, CI: -0.24 - -0.03, P-value: 0.014). Six provinces (KwaZulu-Natal, Gauteng, Limpopo, North West, Mpumalanga, and Western Cape) had a significant association with shedding time.

**Conclusion:**

the duration of viral shedding within our population varies from 1-128 days. Although prolonged shedding might not necessarily indicate infectiousness, individual patient monitoring and management are needed for patients with prolonged shedding. Further studies are required to explore the association between comorbidities and SARS-CoV-2 shedding time.

## Introduction

Coronavirus disease 2019 (COVID-19) is an illness caused by severe acute respiratory syndrome coronavirus 2 (SARS-CoV-2) [1]. The virus was first identified in Wuhan, China in December 2019. COVID-19 has spread globally, causing a global pandemic [2]. On March 5, 2020, South Africa reported its first confirmed COVID-19 case [3]. Despite strict measures such as lockdowns and compulsory wearing of masks put in place to control the epidemic, the virus has continued to spread. Some people remain asymptomatic after acquiring the infection. However, most people develop mild symptoms and recover from the illness without medical intervention [4]. The virus can cause severe disease which requires hospitalization and can be fatal.

During the study period (March - December 2020), three COVID-19 variants (Alpha, Beta, and Delta) were dominant variants of concern that were circulating in South Africa. Currently, there is no effective treatment available for COVID-19 patients. With little known about SARS-CoV-2, clinical trials are being conducted on different treatment options however, there is no strong evidence of improved health outcomes [5]. In 2020, there were no COVID-19 vaccines available for the public in South Africa however, vaccine trials were being conducted [6]. Therefore, estimating SARS-CoV-2 viral shedding time is important for disease prognosis.

Viral shedding time is a useful guide for infectivity and can be used to establish the duration of isolation, and quarantine and inform contact tracing guidelines [7,8]. With the polymerase chain reaction (PCR) test being the best test available for COVID-19 diagnosis, several studies have used it to estimate viral shedding time. The Republic of Korea reported a median shedding time of 15 days among COVID-19 patients [9]. This was different from Japan where the median viral shedding time of COVID-19 patients was reported to be 19 days [10]. Studies show that both symptomatic and asymptomatic patients have the potential to transmit COVID-19 [9-11]. This is evident from a study conducted in Saudi Arabia which found that the mean shedding time of COVID-19 asymptomatic patients was 13.6 days and 16.9 days for symptomatic patients [12]. Factors affecting shedding time include the severity of the disease, age, sex, comorbidity, timeliness of treatment, and COVID-19 symptoms such as fever [7,13,14]. Age and comorbidities have been reported to have an impact on symptom severity and clinical outcome [15].

South Africa has reported the highest number of SAR-CoV-2 infections in Africa. Numerous studies have been conducted globally to estimate the duration of SARS-CoV-2 viral shedding however, no study has estimated viral shedding time for laboratory-confirmed COVID-19 cases in the South African population. Given the rapid spread of the virus, limited information, and treatment options, preventative measures such as quarantine are very important in reducing infections, mortality, and the burden on the health system. South Africa is of particular importance due to its unique epidemiological profile consisting of high HIV/AIDS prevalence, high Tuberculosis incidence, and high death rates from non-communicable diseases such as diabetes.

Since there is no routine test available to measure infectiousness, determining infectiousness, determining viral shedding time, and understanding the associated predictors will play a significant role in informing infection prevention and control measures in COVID-19 patients. This information could be used to understand the association between viral shedding and infectivity and the effectiveness of using a PCR test for infectiousness or re-infection. The results from this study can assist in refining infection control strategies such as clinical management of COVID-19 patients, contact tracing and isolation or quarantine, and in understanding the potential difference of positive PCR tests over time. Clinical management is important in improving health outcomes and quality of care to infected patients. This study aimed to estimate viral shedding time among laboratory-confirmed COVID-19 cases in South Africa.

## Methods

**Study design and procedures:** we performed a cross-sectional analytic study using COVID-19 data obtained from the COVID-19 Notifiable Medical Condition List (NMCList). The study included laboratory results from public and private clinical laboratories in South Africa that reported COVID-19 test results to the NMCList database. The study population consisted of all laboratory-confirmed COVID-19 patients reported to the NMCList database from March to December 2020. We included all laboratory-confirmed COVID-19 patients who had at least two positive PCR test results and subsequently tested negative. The maximum period between the last positive test and the negative test should be 7 days. The person should have done consecutive testing no longer than 14 days between the repeat positive PCR tests. We excluded positive cases that were tested using antigen tests and cases that took a repeat test after 14 days of continuing positivity.

**Data sources:** COVID-19 test results were reported to the NMCList database hosted by the National Institute for Communicable Diseases (NICD). COVID-19 is a category 1 Notifiable Medical Condition (NMC) with a requirement of immediate reporting upon clinical suspicion or laboratory diagnosis followed by a written notification within 24 hours. Clinical data of patients tested for COVID-19 are collected using the NMC reporting platform, which is a national reporting system in South Africa. Both RT-PCR and antigen COVID-19 test results are stored in the NMCList database and reported to the National Department of Health (NDoH). Individuals who have multiple test results are identified using a unique identifier (case_id). Other variables stored in the NMCList include personal identifiers (name, surname, date of birth, sex, national identity number), demographic information (physical address), and health facility information. Data from the NMCList is stored in the NICD surveillance data warehouse. The DATCOV system is an active, prospective sentinel surveillance program for COVID-19 in South Africa. This online platform enables private and public healthcare facilities to submit hospital admissions data for COVID-19 patients. COVID-19 admissions trends are monitored and the epidemiology of the disease in hospitalized patients within the country is described. Admission status (hospitalized or outpatient) was used as a proxy to determine disease severity. Hospitalized patients were defined as patients with severe disease and outpatients as patients with mild disease.

**Data collection:** we extracted data from the NMCList and DATCOV databases. It was assumed that COVID-19 cases that were not in the DATCOV database were not hospitalized. We applied a deterministic method to identify repeat test results from the same individuals and to link patients between the NMCList and DATCOV databases. The patients´ national identity or passport number was used as the linking variable. The linkage helped us determine patients who were admitted and those who were not.

**Case definitions:** COVID-19 case: “a confirmed case of COVID-19 is a person with laboratory confirmation of SARS-CoV-2 infection (using an RT-PCR assay), irrespective of clinical signs and symptoms between March and December 2020” [16]. Negative result: a person with a negative SARS-CoV-2 laboratory test using an RT-PCR assay.

### Variables

**Outcome variable:** the outcome variable is COVID-19 viral shedding time measured in days. Viral shedding time (days) was defined as the period from the date of specimen collection for the first positive PCR test to the date of specimen collection for the last positive test prior to the first negative PCR test. The date of specimen collection for the first positive test was subtracted from the date of specimen collection for the last positive test to obtain the shedding time (in days). The date of onset of viral shedding was defined as the day the specimen of a case was collected since laboratory data had no variable for the date of symptoms onset. This approach catered to individuals who would have been symptomatic or asymptomatic. Explanatory variables included sex, age, province, number of tests done and the severity of COVID-19 (inpatient versus outpatient). The defaults were selected based on the literature.

**Statistical analysis:** data were analyzed using STATA 15 (Version 15, StataCorp, College Station, Texas). Characteristics of study participants were reported using descriptive statistics. Categorical variables (age groups, sex, admission status, and province) were presented using absolute numbers and percentages. Normally distributed continuous variables (age) were reported using means and standard deviations while medians and ranges were used when continuous variables were not normally distributed (shedding time). Initially, simple linear regression was done to assess the association between the dependent variable (shedding time) and each predictor variable. Predictor variables with a p-value of <0.1 from the simple linear regression were added to the multiple linear regression model. We determined differences in shedding time by age, sex, hospital admission status, and province. We reported effect estimates and 95% confidence intervals. We considered a p-value of less than 0.05 as statistically significant.

**Ethical considerations:** the study was conducted in accordance with the Declaration of Helsinki and approved by the Human Research Ethics Committee at the University of the Witwatersrand (Clearance Certificate No. M210443).

## Results

**Figure 1:**
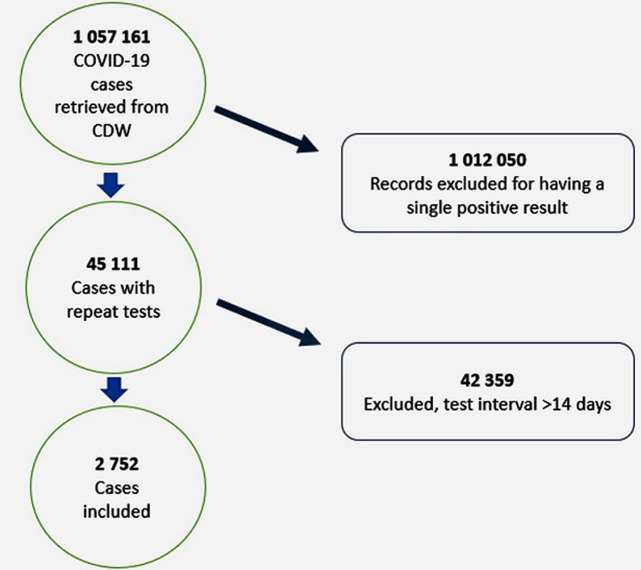
flow chart showing the participant selection process

**Figure 2:**
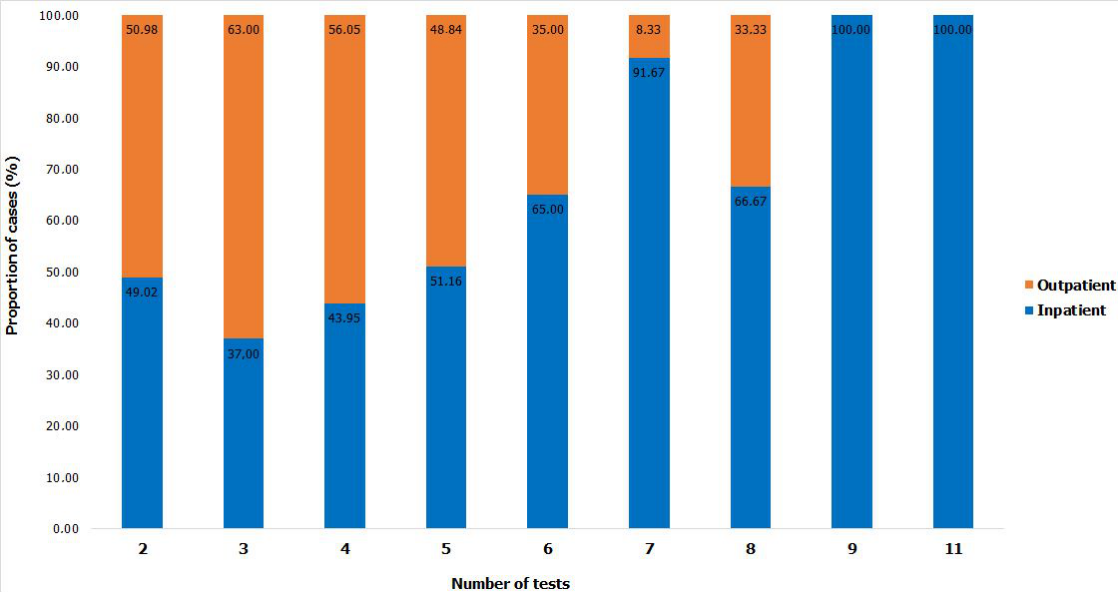
number of positive test results by COVID-19 admission status (inpatients and outpatients) in the study, 2020

**Characteristics of the study population:** there were 1 057 161 COVID-19 cases reported from 5 March to 31 December 2020 in South Africa. Out of these cases, 2752 (0.26%) met the inclusion criteria. Figure 1 shows details of the participant selection process. The 2752 included participants had a total of 8642 PCR positive test results with a median of 3 positive tests per participant (range: 2-11). The majority (74.7%: 2057/2752) of the participants had 3 consecutive positive tests (Figure 2). Patients who had tested 3 times accounted for 75% (2057/ 2752) of the study population. The highest number of tests done was 11 and were done by 1 patient who was an inpatient. Inpatients had the highest numbers of tests conducted compared to outpatients, with 6 tests done by 65%, 7 tests (91.7%), 8 tests (66.6%), and 9 and 11 tests done by inpatients only.

**Shedding time in COVID-19 patients:** Table 1 describes the characteristics of the study population and summarizes the distribution of shedding time by age, sex, province, and admission status. There were more males than females (53% versus 47%). Participants´ ages ranged from 0-99 years, with a mean of 46.4 years (SD: 17.4). Individuals aged 35-44 years were the most frequently represented age group (22.9%), while 0-14-year-olds were the least frequent age group (1.2%). Of all the participants, 39.9% were inpatients and 60.1% were outpatients. Gauteng province had the highest number of participants with 987 (35.9%) cases and Northern Cape had the lowest with 39 (1.4%) cases. The median duration of viral shedding was 17 days, ranging from 1 to 128 days from the date of specimen collection or symptoms onset date. There was a 1-day difference in the median shedding time between males (median: 16 days, range: 1-128) and females (median: 17 days, range: 1-94). Viral shedding time between inpatients (median: 16 days, range 1-108) and outpatients (median: 17 days, range: 1-128) did not differ much either. Also, there was a slight difference in shedding time between age groups. Individuals aged 0-14 years had the lowest shedding time (median: 14 days, range: 1-72) while those aged 25-34 years had the highest shedding time (median: 18 days, range: 1-128). The shortest shedding time was observed in the Eastern Cape with a median of 15 days (range: 1-56) and the longest shedding time was observed in Limpopo with a median of 21 days (range: 1-39).

**Table 1:** baseline characteristics and shedding time of laboratory-confirmed COVID-19 cases in the study, 2020

Variables	Category	Number of cases (n)	Median (range)
**Overall shedding time**		2, 752 (100)	17 (1 - 128)
**Age group (years)**	0 - 14	46 (1.7)	14 (1 – 7)
	15 - 24	172 (6.3)	16 (1 – 56)
	25 - 34	516 (18.8)	18 (1 – 128)
	35 - 44	630 (22.9)	17 (1 – 78)
	45 - 54	547 (19.9)	17 (1 – 84)
	55 - 64	418 (15.2)	15 (1 – 94)
	65+	419 (15.3)	16 (1 – 108)
**Sex**	Male	1,456 (53)	16 (1 – 128)
	Female	1,291 (47)	17 (1 – 94)
**Admission status**	Inpatient	1,099 (39.9)	16 (1 – 108)
	Outpatient	1,653 (60.1)	17 (1 – 128)
**Province**	Eastern Cape	283 (10.3)	15 (1 – 56)
	Free state	70 (2.5)	16 (1 – 67)
	Gauteng	987 (35.9)	17 (1 – 128)
	KwaZulu-Natal	603 (21.9)	17 (1 – 84)
	Limpopo	94 (3.4)	21 (1 – 39)
	Mpumalanga	95 (3.5)	20 (1 – 78)
	North West	94 (3.4)	16 (1 – 54)
	Northern Cape	39 (1.4)	16 (1 – 51)
	Western Cape	487 (17.7)	17 (1 – 84)

In Table 2, we assessed factors associated with shedding time in COVID-19 patients in South Africa. We applied simple and multiple linear regression to compare the association between predictor variables (age, sex, province, and hospital admission status) and SARS-CoV-2 shedding time. Following unadjusted analysis, shedding time did not vary with sex and age group. Regarding the province of residence, the Eastern Cape was considered the baseline for comparisons. Shedding time in Free State and Northern Cape did not differ from shedding time in the Eastern Cape. However, shedding time was longer in KwaZulu-Natal (coefficient: 0.3, CI: 0.1 to 0.5, P-value: <0.001), Limpopo (coefficient: 0.6, CI: 0.3 to 0.9, P-value: <0.001), Mpumalanga (coefficient: 0.5, CI: 0.2 to 0.8, P-value: 0.002), North West (coefficient: 0.4, CI: 0.1 to 0.7, P-value: 0.012) and Western Cape (coefficient: 0.3, CI: 0.1 to 0.5, P-value: 0.001). Shedding time was shorter among hospitalized patients compared to outpatients (coefficient: -0.1: -0.2 to -0.04, P-value: 0.007). After adjusting for age, sex, and province, shedding time remained shorter for hospitalized patients compared to outpatients (coefficient: -0.14, CI: -0.24 to -0.03, P-value: 0.014). Five out of 9 provinces were statistically significant on the univariate and multivariate analysis. The provinces were Gauteng (coefficient: 3.2, CI: 1.7 to 4.7, P-value: <0.001), KwaZulu-Natal (coefficient: 2.6, CI: 1.0 to 4.2, P-value: 0.001), Limpopo (coefficient: 4.1, CI: 1.5 to 6.8, P-value: 0.002), Mpumalanga (coefficient: 4.1, CI: 1.4 to 6.8, P-value: 0.002) and Western Cape (coefficient: 2.8, CI: 1.2 to 4.5, P-value: 0.001). Sex and age were not associated with shedding time.

**Table 2:** univariate and multivariate analysis of factors affecting viral shedding time in COVID-19 patients in South Africa, 2020

Variables		Univariate			Multivariate		
	Category	Coefficient	95%CI	P-value	Coefficient	95%CI	P-value
**Age group (years)**	0 - 14	Ref*					
	15 - 24	-0.2	-0.6 to 0.2	0.376	-1.6	-5.3 to 2.0	0.376
	25 - 34	-0.01	-0.4 to 0.4	0.952	-0.2	-3.6 to 3.2	0.901
	35 - 44	0.1	-0.3 to 0.5	0.696	0.6	-2.7 to 4.0	0.720
	45 - 54	0.03	-0.4 to 0.4	0.870	0.6	-2.7 to 4.0	0.712
	55 - 64	-0.1	-0.5 to 0.3	0.488	-0.3	-3.8 to 3.1	0.848
	65+	-0.1	- 0.5 to 0.3	0.506	-0.3	-3.8 to 3.1	0.854
Sex	Female	Ref*					
	Male	-0.3	-0.1 to 0.07	0.576	-0.4	-1.2 to 0.5	0.403
Admission status	Outpatient	Ref*					
	Inpatient	- 0.1	- 0.2 to - 0.04	0.007	-0.1	-0.2 to -0.03	0.014
Province	Eastern Cape	Ref*					
	Free State	0.2	-0.2 to 0.5	0.339	2.1	0.8 to 5.1	0.158
	Gauteng	0.4	0.2 to 0.6	<0.001	3.2	1.7 to 4.7	<0.001
	KwaZulu-Natal	0.3	0.1 to 0.5	<0.001	2.6	1.0 to 4.2	0.001
	Limpopo	0.6	0.3 to 0.9	<0.001	4.1	1.5 to 6.8	0.002
	Mpumalanga	0.5	0.2 to 0.8	0.002	4.1	1.4 to 6.8	0.002
	North West	0.4	0.1 to 0.7	0.012	2.7	0.05 to 5.3	0.046
	Northern Cape	0.04	-0.4 to 0.5	0.857	0.9	-2.9 to 4.7	0.638
	Western Cape	0.3	0.1 to 0.5	0.001	2.8	1.2 to 4.5	0.001

* p-values – overall p-values for each exposure variable in the model. CI – confidence, intervals univariate analysis, adjusted - multivariable analysis, Ref* - reference category, not included in the final model because it is not significant (p>0.005)

## Discussion

This study aimed to estimate the viral shedding time among laboratory-confirmed COVID-19 cases in South Africa. Understanding SARS-CoV-2 shedding time is important for determining effective public health interventions such as the duration of isolation and wearing of masks. Viral shedding can be used as a proxy for infectivity, therefore is important for the clinical management of COVID-19 cases. We found that the median shedding time in our sample was 17 days from the symptom onset date or date of specimen collection. Shedding time ranged from 1 to 128 days. Our study showed a 1-day difference in the median shedding time between males and females and between inpatients and outpatients. We also found a slight difference in shedding time between age groups.

A study conducted on the Canadian population [17] found that the median shedding time was 19 days which is 2 days more than our findings among the South African population. Another study conducted in the United States of America reported a median shedding time of 21 days within their population, which is higher than the shedding time we found within our population [18]. The difference in shedding time between South Africa, Canada, and the United States could be due to the difference in population characteristics in these geographic locations. Our findings are similar to those reported in a study conducted in China which found that the median shedding time among adults was 17 days [7]. Although our study shows a similar median shedding time to that of China, our study included both children and adults and covered a wider population whereas the study conducted in China only included adults. The difference in viral shedding across regions could be attributed to new mutations of COVID-19. The mutations can affect disease severity, symptoms, and effectiveness of treatments and increase the risk of infection [19]. Our study is supported by another study that found no significant difference in the median shedding time between males and females and further reported that prolonged shedding was not affected by signs and symptoms of COVID-19 [7]. Another study conducted on outpatients and inpatients in the United States of America found that the viral shedding time of inpatients was similar to that of outpatients [18].

Age group 0-14 years had a median shedding time of 14 days which was the lowest among other age groups. Age group 25-34 reported the highest shedding time with a median shedding time of 18 days. The slight difference in shedding time between age groups might be because people live in families and communities where people of different age groups live together, allowing for transmission to happen within different age groups. Our study found that South African children had a lower shedding time compared to children in South Korea who were reported to have an average shedding time of 17.6 days [20]. Another study found that children less than 18 years old had a shorter shedding time compared to other age groups [21]. The study further highlights that viral shedding can happen in children presenting with symptoms and those without symptoms, with a shedding time of up to 3 weeks (21 days).

Children and young adults are more likely to have mild disease and less likely to have severe or fatal COVID-19 compared to adults [22]. The age-related difference in the severity of COVID-19 in children and adults could be because children have a lower intensity of exposure to SARS-CoV-2. Furthermore, it is thought that children have a stronger innate immune system, which acts as the first line of defense against SARS-CoV-2 [20]. In general, testing patterns in children are different from those in adults, and children are most often tested when they are symptomatic or a contact of a case. SARS-CoV-2 is shed when talking, coughing, sneezing, or even exhaling. Studies also show that the virus can be shed via faeces and urine and can continue for several weeks [23,24]. This makes it possible for the virus to spread in schools where they share classrooms and objects and in pre-schools where children are taken care of by the same teacher.

Viral shedding can continue for longer periods in some patients. Two cases had a shedding time greater than 100 days. The cases were a 77-year-old inpatient male who shed the virus for 108 days and a 34-year-old outpatient male who shed the virus for up to 128 days. A study of 38 COVID-19 patients with prolonged shedding reported that the longest shedding time they observed was 118 days and was a 77-year-old male [25]. Although we could not determine whether these patients had symptoms or not, research shows that patients with prolonged shedding presenting with mild disease have the potential of experiencing a reemergence of symptoms a week or more after initial symptoms have resolved [26]. It is difficult to determine why certain individuals have such prolonged shedding and further research is needed to better understand this. A study conducted on six COVID-19 patients shows that prolonged viral shedding can be observed in both asymptomatic and severe patients [25]. Severe disease, comorbidities, socioeconomic status, old age, and male sex have been reported as factors associated with prolonged SARS-CoV-2 viral shedding [13,17,27,28]. In line with another study, our study found no association between age and shedding time [29]. However, some studies have reported that older age is associated with prolonged shedding [30,31].

Older adults usually have a higher prevalence of comorbidities, such as obesity, hypertension, and heart disease, which are associated with severe COVID-19 compared to children [15]. Although children can also experience severe clinical disease, they are less affected by COVID-19 than adults. Other studies have reported prolonged shedding; however, prolonged shedding was mainly reported in patients with severe disease [30,32]. The United States Centers for Disease Control and Prevention (CDC) recommended that people with possible COVID-19 exposure should quarantine for 14 days and people who test positive should isolate for 10 days from the date of symptoms onset [33]. South Africa however recommended 10 days of isolation or quarantine for asymptomatic patients, and patients with mild, moderate, or severe disease [34]. Of the people infected with COVID-19, only 20% experience moderate or severe disease and require hospitalization. Eighty percent experience mild COVID-19 and isolate from home. Also, it has been reported that asymptomatic or pre-symptomatic cases are the main drivers of COVID-19, accounting for about 50% of new COVID-19 infections [27].

Therefore, it is important to monitor viral shedding in outpatients or patients with mild disease because they can shed the virus longer and could potentially be a source of future COVID-19 outbreaks. Our findings indicate that infection control strategies provided by the CDC and the NDoH should take into account prolonged shedding and its impact on disease transmission. Our study found a significant association between inpatients and shedding time. Inpatients had 1 day less shedding time compared to outpatients. Based on our results, it is important for patients with mild disease or asymptomatic patients to follow public health interventions such as quarantine and wearing of masks as recommended. Studies show that COVID-19 patients who are hospitalized usually have COVID-19 symptoms, and comorbidities and are more likely to be older [35-38]. It was reported that COVID-19 patients with comorbidities such as HIV, chronic obstructive pulmonary disease (COPD), chronic obstructive pulmonary disease (COPD), and diabetes are more likely to develop severe disease and have an increased risk of mortality. The presence of comorbidities increases viral clearance by up to 4 days compared to those without comorbidities [39]. These findings show that early interventions in COVID-19 patients with comorbidities could reduce morbidity and mortality.

Several studies have reported no association between viral shedding time and sex [30,40,41]. The association between shedding time and province varied. In our study, univariate and multivariate analysis results showed that KwaZulu-Natal, Gauteng, Limpopo, North West, Mpumalanga, and Western Cape had a significant association with shedding time. The differences in shedding time between different regions may be due to differences in comorbidities, socioeconomic status, type of test used, sample size, and disease severity.

The strengths of our study include the following: We used data from the whole population with COVID-19 to estimate shedding time. Also, we included both inpatients and outpatients which allowed us to estimate the difference in shedding time within the two groups. We only used test results that were tested using PCR-test which is the gold standard. This is essential for accurate results thus ensuring that negative tests were correctly identified therefore giving accurate estimates of shedding duration. With limited treatment options during the study period, our results may be used to understand the viral shedding duration and factors associated with viral shedding. This information may be used to strengthen surveillance and facilitate early intervention which will reduce disease transmission.

Our study had some limitations. Firstly, data received from the NMCList did not include clinical information about the cases. Secondly, due to the secondary nature of the study, we could not get certain variables that could have assisted in determining other factors affecting shedding time within the population. Also, we could not analyze the impact of vaccines and treatment on shedding time. Thirdly, due to the exclusion of patients with a single positive result, selection bias could have been introduced, which might have also affected external validity, thereby potentially compromising the generalizability of our findings. Lastly, our study was retrospective and contained a relatively small sample size however, the results may be useful in understanding the transmission dynamics of COVID-19. Therefore, we recommend that a similar study should be conducted on a larger sample. Viral shedding before symptom onset and before testing was not measurable using the data we obtained. It is likely that viral shedding occurs beyond the time points we have described. It is also possible that the shedding time presented in our study is likely to be an underestimate of the true shedding time in the population.

## Conclusion

Our study found a significant association between the hospital admission status of COVID-19 patients and shedding time. There was a 1-day difference in shedding time between inpatients and outpatients. Therefore, there is a need to strengthen prevention and control strategies such as contact tracing as both inpatients and outpatients can shed the virus for a similar duration. We discovered that factors such as age and sex were not associated with shedding time. The duration of viral shedding within the population of South Africa varies from 1 to 128 days. Although prolonged shedding might not necessarily indicate infectiousness, individual patient monitoring and management are needed for patients with prolonged shedding. Furthermore, to control SARS-CoV-2 infection, different approaches to effectively address transmission risk in asymptomatic patients are required.

Our study only looked at four predictor variables (sex, admission status, age and province). We recommend that further studies be conducted, including more variables such as comorbidities, socio-economic status, level of education and vaccination status. Since continuous positivity might not necessarily mean infectivity, we recommend that future studies consider evaluating live viruses within the population and do a follow-up on cases with prolonged shedding to assess their contacts. This can assist in figuring out infectivity and can be used to inform isolation and quarantine requirements. We also recommend that studies evaluate the impacts of possible symptom recrudescence on shedding time and its impacts on SARS-COV-2 transmission.

### What is known about this topic

- *COVID-19 is an infectious disease transmitted from person to person by small droplets or contact with contaminated surfaces*;
- *COVID-19 can mutate, causing variants that could be more infectious and could lead to severe disease*.

### What this study adds

- *This study reports on viral shedding time in COVID-19 patients in the South African population in 2020 before the introduction of the COVID-19 vaccines*;
- *The study shows factors associated with shedding time in the population*;
- *The findings in this paper could assist other researchers to understand the transmission dynamics of SARS-CoV-2 in a low-income country and use the information for public health interventions*.
